# Acellular Gelatinous Material of Human Umbilical Cord Enhances Wound Healing: A Candidate Remedy for Deficient Wound Healing

**DOI:** 10.3389/fphys.2017.00200

**Published:** 2017-04-04

**Authors:** Nazihah Bakhtyar, Marc G. Jeschke, Laurence Mainville, Elaine Herer, Saeid Amini-Nik

**Affiliations:** ^1^Department of Biological Sciences, Sunnybrook Health Sciences Center, Sunnybrook Research InstituteToronto, ON, Canada; ^2^Division of Plastic Surgery, Department of Surgery, University of TorontoToronto, ON, Canada; ^3^Department of Gynecology and Obstetrics, Sunnybrook Health Sciences Centre, University of TorontoToronto, ON, Canada; ^4^Department of Laboratory Medicine and Pathobiology, University of TorontoToronto, ON, Canada

**Keywords:** skin, wound healing, deficient healing, umbilical cord, Wharton's jelly, stem cell, regenerative medicine, tissue regeneration

## Abstract

Impaired wound healing is a severe clinical challenge and research into finding effective wound healing strategies is underway as there is no ideal treatment. Gelatinous material from the umbilical cord called Wharton's jelly is a valuable source of mesenchymal stem cells which have been shown to aid wound healing. While the cellular component of Wharton's jelly has been the subject of extensive research during the last few years, little is known about the de-cellularized jelly material of the umbilical cord. This is important as they are native niche of stem cells. We have isolated Wharton's jelly from umbilical cords and then fractionated acellular gelatinous Wharton's jelly (AGWJ). Here, we show for the first time that AGWJ enhances wound healing *in vitro* as well as *in vivo* for wounds in a murine model. *In vivo s*taining of the wounds revealed a smaller wound length in the AGWJ treated wounds in comparison to control treatment by enhancing cell migration and differentiation. AGWJ significantly enhanced fibroblast cell migration *in vitro*. Aside from cell migration, AGWJ changed the cell morphology of fibroblasts to a more elongated phenotype, characteristic of myofibroblasts, confirmed by upregulation of alpha smooth muscle actin using immunoblotting. AGWJ treatment of wounds led to accelerated differentiation of cells into myofibroblasts, shortening the proliferation phase of wound healing. This data provides support for a novel wound healing remedy using AGWJ. AGWJ being native biological, cost effective and abundantly available globally, makes it a highly promising treatment option for wound dressing and skin regeneration.

## Introduction

A wound is described as a disruption of the normal healthy anatomical structure and functional integrity of the skin (Atiyeh et al., 2002). The wound healing process involves four integrated and overlapping phases: hemostasis, inflammation, proliferation and tissue remodeling or resolution (Gosain and DiPietro, 2004). These steps allow for the reformation and revascularization of the skin by allowing a functional dermis and epidermis to form. About 1.5 billion people suffer from skin diseases as a consequence of both progressive aging and the lack of adequate health-care (Valacchi et al., 2012). Among these, skin lesions are highly prevalent and can be divided into acute or chronic wounds (Whitney, 2005). It has been reported that acute wounds generally follow trauma or inflammation and usually heal within 6 weeks (Valacchi et al., 2012). In certain circumstances, chronic wounds, also known as non-healing wounds result which do not progress through the normal wound healing steps and fail to heal within 6 weeks, this type of wound results in an open laceration of varying degrees of severity (Branski et al., 2009). Such wounds often enter a state of pathologic inflammation due to a postponed, incomplete, or uncoordinated healing process. The management of a chronic wound defined as a barrier defect that has not healed in 3 months—has become a major therapeutic challenge throughout the western world, and it is a problem that will only worsen since the incidence of conditions that prevent wound healing, such as diabetes, obesity, vascular disorders are on the rise in addition to burn injuries (Nunan et al., 2014).

The primary goals in the treatment of wounds are rapid wound closure and a functional and esthetically satisfactory scar. In order to do so, wound coverage is one of the cornerstones of wound management (Singer and Clark, 1999). Clinicians may use various materials and techniques, such as antibiotic therapy, surgical debridement, negative pressure devices, wound dressings, hyperbaric oxygen therapy, antimicrobial therapy, bioengineered skin equivalents and growth factors but these have limited success which illustrates the complexities of wound healing. Skin grafts from allogeneic or autologous sources, reconstructive tissue flaps, and engineered skin substitutes can also be useful in the treatment of more extensive or chronic wounds (Valacchi et al., 2012). Although research continues and skin substitutes gain in efficacy, wound healing remains a clinical challenge, particularly with an aging population (Jeschke et al., 2015, 2016). Clinical and pre-clinical studies using cell-based therapies are being gradually introduced into medical care to manage skin wounds because they can repair/replace damaged tissue with a healthy tissue due to their natural ability to produce cytokines and molecules necessary for wound healing (Marfia et al., 2015; Markeson et al., 2015; Nicholas et al., 2016a,b).

In 1991 McElreavey et al. first isolated the mesenchymal stromal cells from the Wharton's jelly portion of the umbilical cord (McElreavey et al., 1991). Wharton's Jelly is a source of perinatal mesenchymal stem cells (WJ-MSC) with unique properties of both embryonic and adult stem cells (Pirjali et al., 2013). Wharton's jelly-derived MSCs have the ability to maintain phenotypic attributes, cell growth kinetics, cell cycle pattern, *in vitro* multilineage differentiation plasticity, apoptotic pattern, normal karyotype-like intrinsic MSC properties in long-term *in vitro* cultures (Sabapathy et al., 2014). Several studies have investigated cellular therapy with WJ-MSCs, alone or within a scaffold (Azari et al., 2011; Shohara et al., 2012; Fong et al., 2014; Ribeiro et al., 2014; Sabapathy et al., 2014). Studies have elucidated the role of WJ-MSCs for various applications, such as for neurological disorders (Shalitin et al., 2003), kidney injury (Du et al., 2013), lung injury (Moodley et al., 2009), liver injury and cancer therapy (Sabapathy et al., 2014). We have recently reported that WJ-MSCs conditioned-medium with its secretome has positive effects on wound healing *in vitro* (Arno et al., 2014).

Mesenchymal stem cells in Wharton's jelly are in close interaction with their extra cellular matrix. Considering that the secretome of WJ-MSCs enhances wound healing *in vitro*, we asked whether the native extracellular matrix of these cells, which is not necessarily secreted by WJ-MSCs, have any effect on skin healing. The Wharton's jelly itself without cells has not been investigated with regards to wound healing. Wharton's jelly contains a significant amount of extracellular matrix components which are composed primarily of collagen, hyaluronic acid, and various sulphated proteoglycans. It is well-known that biosynthesis of extracellular matrix components is enhanced by several peptide growth factors, mainly insulin-like growth factor (IGF) (Edmondson et al., 2003), fibroblast growth factor (FGF) (Yu et al., 2003) and transforming growth factor b (TGF-b) (Shalitin et al., 2003). These growth factors may accumulate within Wharton's jelly to support the cells (e.g., MSCs). We, therefore, hypothesized that acellular gelatinous Wharton's jelly (AGWJ) enhances skin wound healing. Here we report on the effects of AGWJ treatment on fibroblast cells of the dermis through *in vitro* experiments. We then performed wound healing experiments on 8 weeks old male C57/black 6 mice to establish the effect of AGWJ treatment on wounds *in vivo*. The resulting data support the investigation for a novel wound healing treatment using AGWJ.

## Materials and methods

### Cell culture

Tissue culture plastic ware were purchased from BD Falcon™ (Bedford, MA, USA), and all tissue culture media and supplements were purchased from Wisent Inc. (St-Jean-Baptiste, QC, Canada), unless otherwise stated. Fibroblast cell culture medium was high-glucose Dulbecco's modified Eagle's medium (DMEM) supplemented with 10% fetal bovine serum (FBS) and 1% antibiotic–antimycotic solution. Primary human normal skin fibroblasts were obtained from skin tissue samples. The skin was dissected to remove any underlying fat from the dermis, cut into small explant pieces of 2 to 4 mm, and cultured in 10 cm dishes. When fibroblast cells had migrated out of the tissue and onto the plate, the tissue pieces were removed. When fibroblasts reached to 70% confluence, approximately within 1 week, they were trypsinized with 0.05% trypsin in preparation for subculture. Fibroblasts were subcultured in 75 cm^2^ tissue culture flasks at a density of 5000 cells/cm^2^.

### Acellularization of wharton's jelly

Sterile umbilical cords were obtained from cesarean sections performed by surgeons from the department of Gynecology and Obstetrics at Sunnybrook Health Sciences Center after obtaining consent from the patients. The cords which were on average 20 cm in length were cut in half. The 10 cm half cords were then cut open lengthwise to reveal the Wharton's jelly contents. The cord pieces were then washed by dipping them in a 2% antibiotic/antimycotic PBS solution followed by dipping them in a 1% antibiotic/antimycotic PBS solution. The cord was then opened completely and a sterile scalpel was used to scrape the jelly out of the umbilical cord. The jelly (approximately 5 ml) was then placed in a 50 ml conical tube with 20 ml of 1× DMEM complete media and resuspended vigorously using a 5 ml pipette to break apart the jelly. The resuspended jelly in media was then centrifuged at 1400 rpm for 10 min. The cells and cord debris were pelleted. The supernatant with AGWJ was collected and frozen at −80°C. The cell pellet was discarded (Supplemental Figure 1).

### Cell migration study: scratch assay

Normal human skin fibroblasts were seeded in six well plates at a cell density of 20,000 cells/well. When the wells had reached to 100% cell confluency, two scratches were made with a 1000 μl pipette tip. The media was then aspirated and the cells were washed with PBS. The PBS was aspirated. One well was immediately stained with crystal violet as a 0 h time point. Treatment media of AGWJ in DMEM or complete DMEM control media was added to the cells. After 24 h, the cells were stained with crystal violet. Images were taken on a microscope (Zeiss) with 4× magnification. Eight images were taken per scratch. Quantification was performed using ImageJ software (National Institutes of Health, Bethesda, MD, USA). The cells within the scratch area were counted as the cells in scratch zone.

### *In vivo* wound healing model

Eight C57/black 6 (8 weeks old, male, body weight 25 to 30 g) were obtained from Jackson Laboratory under the guidelines of the Sunnybrook Research Institute and Sunnybrook Health Sciences Animal Policy and Welfare Committee of the University of Toronto. Animal procedures were reviewed and approved by Sunnybrook Research Institute and Sunnybrook Health Sciences Centre at University of Toronto animal care and use committee. Animals were anesthetized and back cutaneous hair was removed by electrical shaving under anesthesia as stated in the Animal Protocol. 4 wounds of 6 mm diameter full-thickness skin wounds were created on each side of the midline. The animals were randomly divided into two groups: treatment (AGWJ and Matrigel; Corning Matrigel matrix high concentration product#354262 Corning, NY, USA) and control (complete DMEM medium and Matrigel). For the 5 day time point study: 3 mice received control treatment on their wounds and 3 mice received AGWJ treatment. For the 7 day time point study: 6 mice received control treatment and 7 mice received AGWJ treatment. Each wound topically received 100 μl AGWJ treatment or control DMEM in Matrigel mix. The day of the wounding was counted as day 0. For the 7 day time point study, on days 2 and 4 the wounds were redressed. On day 6, 24 h before sacking the mice, the animals received an intraperitoneal injection of bromodeoxyuridine (BrdU) (Calbiochem, San Diego, CA, USA). For the 5 day time point, the wounds were redressed on day 2, BrdU injected on day 4 and the mice were sacrificed on day 5.

### Ethical regulations for animal study

The animal experiments were reviewed and approved by, and performed in accordance with the guidelines and regulations set forth by the Sunnybrook Research Institute and Sunnybrook Health Sciences Animal Policy and Welfare Committee of the University of Toronto, Ontario Canada. All procedures using animals were approved by the Sunnybrook animal care committee, approval #15-503(M-1) issued Nov 20, 2015 under the auspices of Canadian Council on Animal care.

Human umbilical cords were obtained from cesarean sections performed by surgeons from the Department of Gynecology and Obstetrics at Sunnybrook Health Sciences Center, University of Toronto, Toronto, Ontario, Canada. All subjects gave written informed consent in accordance with the Declaration of Helsinki Principles. The protocol was approved by Toronto Academic Health Sciences Network (TAHSN) and University of Toronto-affiliated Sunnybrook Research Institute and Sunnybrook Health Sciences Centre Institutional Ethics Review Board approval (REB number: 017-2011 valid until March 1, 2017), and after getting patient signed informed consent.

### Wound analysis

On day 5 and day 7 the mice were sacrificed. The wounds were then excised using a scalpel and surgical scissors. 3 out of the 4 wounds were placed in histology cassettes and fixed in 10% buffered formalin for 24 h at 4° and then switched to a 70% ethanol solution and then embedded in paraffin. Specimens were cut into 5 μm sections. Tissue specimens were cut through the center or midline of the wound, providing a cross section through the wound center. Both halves of the wound were then placed on slides for analysis. The wounds also included some normal intact skin on either side of the wound. The 4th wound was frozen immediately after excision for future analysis purposes. For high power field (HPF) analysis of wounds, granulation tissue was selected from the center of the wound which was the healed area from both the control and AGWJ treated wounds.

### Trichrome stain

Trichrome reagents were from EMS (Hatfield, PA, USA) unless otherwise stated. Paraffin-embedded slides were heated for 30 min at 60°C. Slides were then deparaffinized with citrosol, followed by rehydration through 100% × 2, 95%, 70% and washed in distilled water. Slides were placed in Bouin's solution (26367–01; EMS, Hatfield, PA, USA) overnight at room temperature overnight and rinsed under running tap water for 10 min. Hematoxylin stain (HHS16; Sigma, Saint Louis, MO, USA) and Biebrich scarlet-acid fuchsin solution were applied sequentially for 10 min. Washes were performed after each stain addition. Slides were differentiated in phosphomolybdic–tungstic acid for 15 min, and were transferred to aniline blue for 5 min. Slides were next rinsed and differentiated in 1% acetic acid for 2 min then washed in distilled water. Slides were then dehydrated through 95% ethanol and absolute ethanol followed by clearing in citrosol. Slides were mounted with SHUR/Mount xylene-based liquid mounting media (Triangle Biomedical Sciences, Durham, NC, USA). Images were acquired using a Zeiss Axiovert 200 light microscope at 5×, 10×, 20×, and 40× magnification. Quantification was carried out using merged images to measure the entire wound length.

### Immunohistochemistry

Paraffin-embedded skin tissue slides were deparaffinized by heating for 30 min at 60°. Slides were then placed in citrosol (2×), 100% Ethanol (2×), 95% ethanol, 70% ethanol for 3 min each, followed by water. Antigen retrieval was then performed using antigen decloaker (1×; Biocare Medical, Concord, CA, USA) which was added to the slides in a preheated decloaking chamber for 4 min at 110°C. For BrdU staining, samples were denatured with 1.5 N HCl for 30 min at 37°C and neutralized with 0.1 M borate buffered twice for 5 min. Samples were blocked with 3% H_2_O_2_ for 10 min, and then washed with washing buffer (0.05 M Tris–HCl, 0.15 M NaCl, 0.05% Tween 20 in deionized water). The primary antibody (mouse monoclonal anti-BrdU, 1:200; Cell Signaling, Beverly, MA, USA) was diluted in PBS and incubated at room temperature for 1 h. For α smooth muscle actin (α SMA) analysis, a different section was probed with αSMA (mouse monoclonal anti-αSMA, 1:200; clone 1A4; ebioscience, Sandiego CA, USA) primary antibody diluted in PBS was incubated at room temperature for 1 h. Slides were then incubated for 15 min with MACH3 mouse probe (Biocare Medical), followed by MACH3 rabbit or mouse horseradish peroxidase polymer, with washes in between. The betazoid diaminobenzidine chromogen kit (Biocare Medical) was mixed and added for 5 min or until brown stain was noticeable. The reaction was terminated with running water. Nuclear staining was carried out with hematoxylin for 30 s, followed by differentiation with three dips in 1.5% acid alcohol and bluing in 0.1% sodium bicarbonate for 10 s. Sections were dehydrated through 95% and absolute ethanol to citrosol and mounted with SHUR/Mount. Images were acquired using a Zeiss Axiovert 200 light microscope at 10× magnification to image the whole section followed by 40× magnification to further focus on the wound margins and the wound center. The 40× magnification images were quantified by positive cells using ImageJ software, and then normalized to the number of total cells in the 40× field.

### Immunofluorescence

Cells were washed with phosphate-buffered saline (PBS) and fixed for 15 min in 4% paraformaldehyde (Alfa Aesar, Karlsruhe, Germany). Fixed cells were washed in PBS and permeabilized for 10 min with 0.5% Triton X-100 solution in PBS. Cells were washed again and then blocked for 30 min with 1% bovine serum albumin in 0.5% Triton X-100 in PBS. A mouse monoclonal αSMA (anti-αSMA, 1:200; clone 1A4; ebioscience, Sandiego CA, USA) primary antibody was added and incubated overnight at 4°C. After washing with PBS, the secondary antibody was added in 1% bovine serum albumin in 0.5% Triton X-100 in PBS and incubated for 1 h at room temperature in the dark (Alexa Fluor 488 donkey anti-mouse, 1:500; Life Technologies, Eugene, OR, USA). The cells were then washed three times with PBS, the slides were mounted with Vectashield mounting medium with 4′,6-diamidino-2-phenylindole (DAPI; Vector Laboratories, Burlingame, CA, USA). Cells were examined and photographed using an Apotome Axiovert fluorescent imaging system at 10× magnification (Zeiss, Oberkochen, Germany). Four images were taken per well and two wells were imaged, one well was AGWJ treated cells and one was control treated cells.

### Cell viability assay

Cell viability was performed using the CellTiter-glo luminescent cell viability assay kit (G7571 Promega, Madison, WI, USA). Briefly, cells were seeded into a 96 well plate and allowed to grow for 24 h at 37°C in a 5% CO2 incubator. After 24 h, the media was aspirated and AGWJ treatment or control media was added to the cells. The plate was again incubated for 24 h in the incubator. After the 24 incubation with treatment, the cell titer glo substrate was added to the buffer and then added to the plate. The cell titer glo solution was added in a 1:1 ratio to the volume of media in the wells. The plate was shaken to mix the solution with the media and then incubated for 10 min at room temperature. After which the luminescence was read using the Synergy H4 hybrid multi-mode microplate reader (100 Tigan Street Winooski, VT, USA).

### Cell lysate preparation

Cell lysates were prepared on ice. Media was aspirated from the cells. The cells were washed with PBS, the PBS was then aspirated. Next, lysis Buffer (RIPA) with 5 mM EDTA, 1× protease inhibitor, and phosphatase inhibitor was added to the cells. 150 ul of lysis buffer was added to a 10 cm plate of cells. The cells were scraped off the plate with a cell scraper. The solution was placed in an eppendorf tube and incubated on ice for 30 min with vortexing every 10 min. The cell lysate was then centrifuged at 4°C for 30 min at 20,000 × g. The supernatant was collected in an eppendorf tube and stored at −80°C.

### Western blot analysis

Briefly, cell lysates (15 μg of protein per well) were separated by 10% SDS-PAGE gel, proteins were then transferred to nitrocellulose membrane, after which the blots were blocked with 5% skim milk in TBST buffer. The blots were washed three times in TBST buffer and then blots were probed using the mouse monoclonal αSMA (anti-αSMA, 1:1000; clone 1A4; ebioscience, Sandiego CA, USA), mouse monoclonal vimentin (anti-vimentin, 1:1000; Thermofisher Scientific, Waltham MA, USA). Loading control used was GAPDH (anti-GAPDH, 1:5000, Cell Signaling, Danvers MA, USA). Primary antibody incubated overnight at 4°C. Band intensities were detected, normalized and quantified with the Chemidoc and Image Lab 5.0 software (Bio-Rad Laboratories).

### Statistical analysis

The statistical comparisons between the AGWJ treatment and control groups were performed using a 2-tailed Student's *t*-test for analysis. A *P* value of <0.05 was considered statistically significant. Data were graphically represented as the mean of the target group ± the 95% confidence interval. Microsoft excel was used for data analysis.

## Results

### AGWJ treatment enhances wound healing in mice

**Figure 1:**
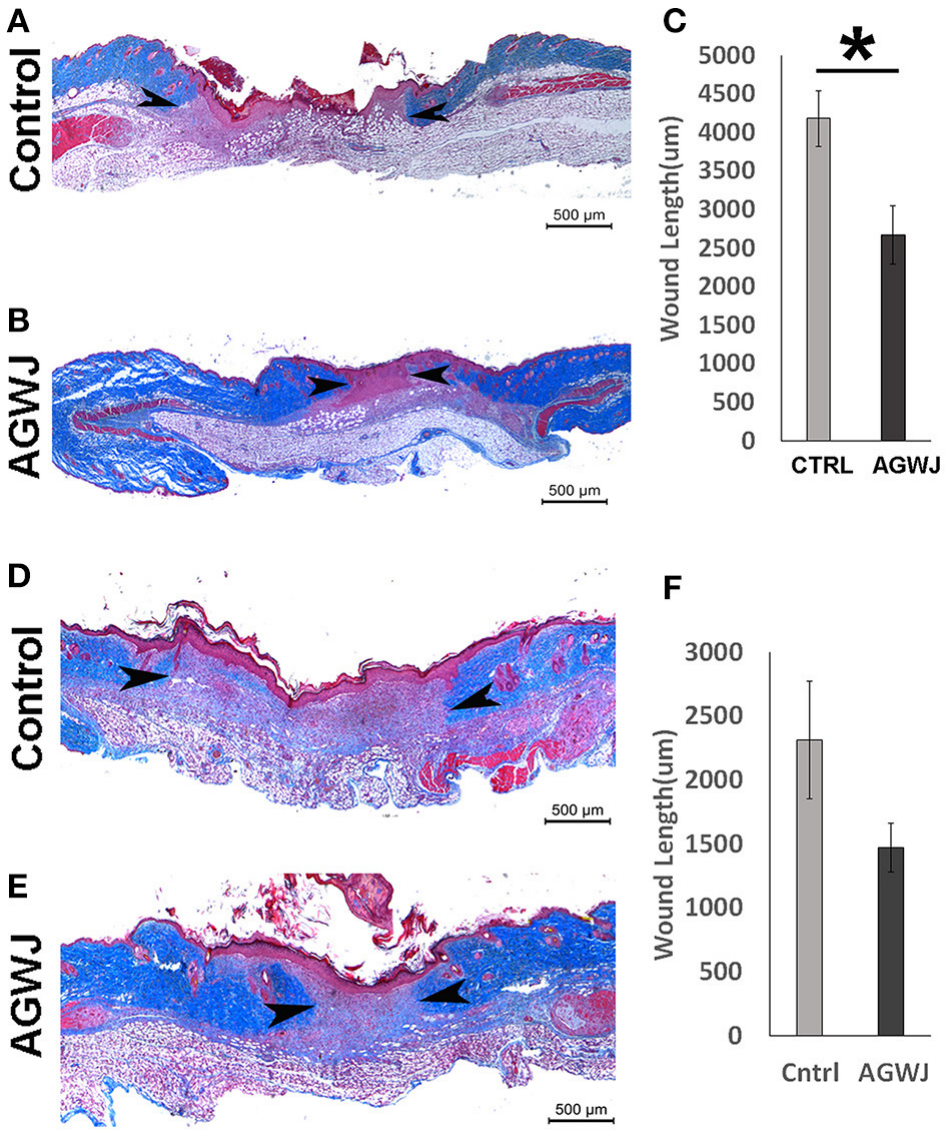
**Effect of AGWJ treatment on wound healing ***in vivo*** after 5 days and 7 days**. Wound length was assessed using trichrome staining. **(A)** The representative image displaying the length of a control wound after the 5 day wound healing study. **(B)** A representative AGWJ treated wound displaying the healing at the end of the 5 days. **(C)** Quantitative analysis of the trichrome stain results comparing the wound length between control and AGWJ treated wounds. **(D)** The representative image displaying the length of a control wound after the 7 day wound healing study. **(E)** A representative AGWJ treated wound displaying the healing at the end of the 7 days. **(F)** Quantitative analysis of the trichrome stain results comparing the wound length between control and AGWJ treated wounds. Data shown are mean ± 95% confidence interval. *P*<0.05 compared with control. *N* = 3 for AGWJ and *N* = 3 for control treated mice for the 5 day study. *N* = 6 for control mice for 7 day study, *N* = 3 for treatment mice, each N represents one animal. Black arrow head shows the border of the wound and intact skin. ^*^Indicates the significance of *p* < 0.05 compared to control.

To validate if there are any beneficial effects of AGWJ on promoting wound healing *in vivo*, we performed two wound healing studies on a murine model. We conducted a 5 day and a 7 day time point study. Wound length and hence wound closure were analyzed using trichome staining on excised wounds after the end of both studies. The results showed that after 5 days, compared with the control treatment (Figure 1A), AGWJ treatment led to a significant reduction in the wound size (Figure 1B). Wound length was measured from the border of the intact skin on the right side through the wound bed to the intact skin on the left side of the wound. All of the wounds were measured and the average wound length for the control treated wounds was 4184.09 um ± 358.18 and for the AGWJ treated wounds, the average wound length was 2673.53 um ± 379.02. ^*^P < 0.05 (Figure 1C). For the 7 day treatment time point, the results also display a dramatic reduction in the wound length with AGWJ treatment as compared with controls, however, the data does not show significance (Figures 1D–F). These results show that AGWJ treatment promotes enhanced wound healing observed through shorter wound length and hence faster wound closure at the proliferation phase of skin healing in young mice.

### AGWJ treatment does not affect cell proliferation or cell viability of fibroblasts

**Figure 2:**
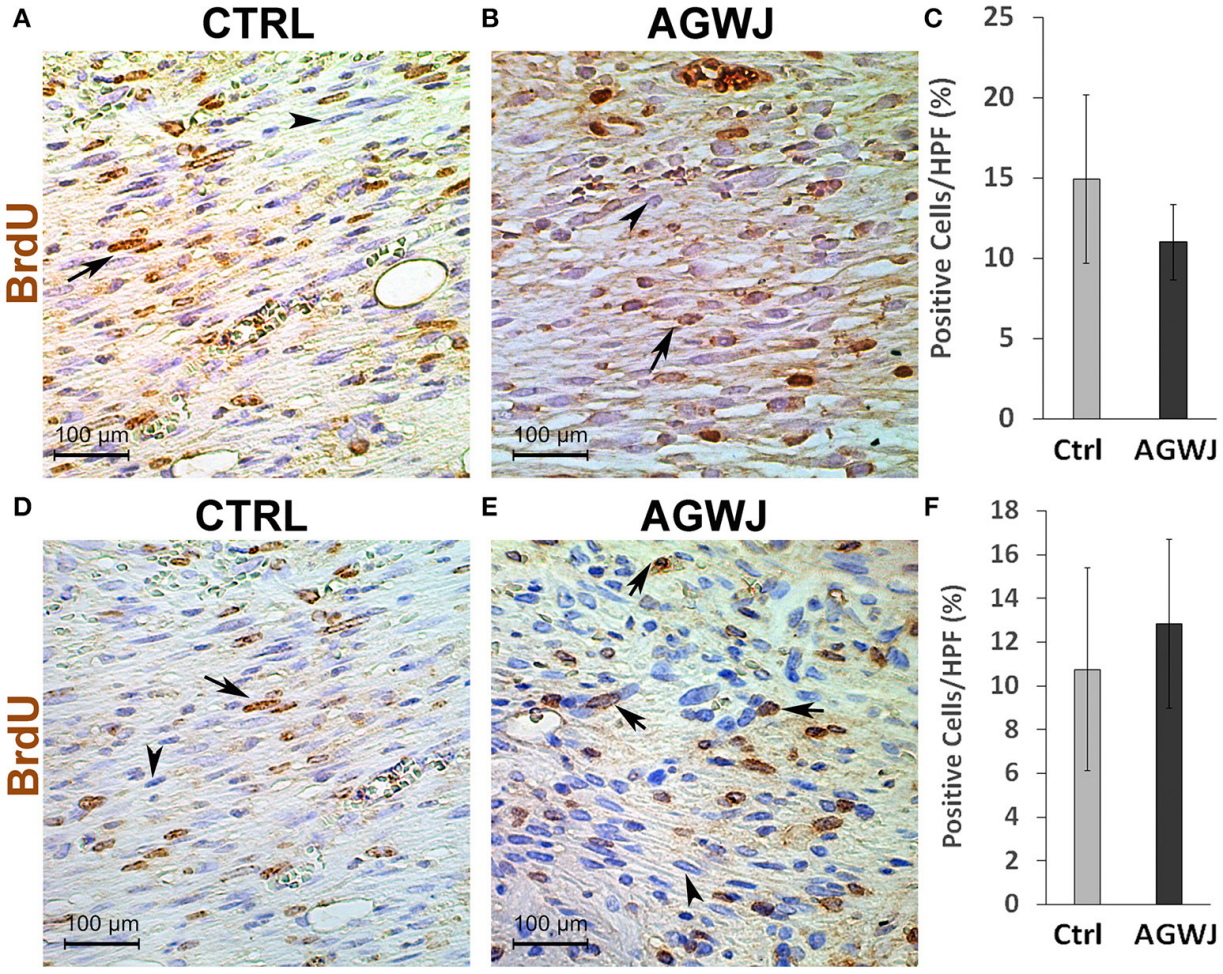
**BrdU analysis of cell proliferation in the wound bed after AGWJ treatment compared to controls. (A)** Representative IHC image displaying BrdU-positive cells in the wound bed of a control treated wound after 5 day time point. **(B)** BrdU-positive cells in an AGWJ treated wound after 5 day time point. **(C)** Quantification of BrdU-positive cells in the wound bed after AGWJ treatment for 5 day time point compared with control treatment. The graph shows the percentage of BrdU positive cells per high power field (40×). **(D)** Representative IHC image displaying BrdU-positive cells in the wound bed of a control treated wound after 7 day time point. **(E)** BrdU-positive cells in an AGWJ treated wound after 7 day time point. **(F)** Quantification of BrdU-positive cells in the wound bed after AGWJ treatment for 7 days compared with control treatment. The graph shows the percentage of BrdU positive cells per high power field (40×). Data shown are mean ± 95% confidence interval. For the 7 day study, *N* = 7 for AGWJ treated mice and *N* = 6 for control mice. For the 5 day study, *N* = 3 for AGWJ and *N* = 3 for control treated mice. Each N represents one animal. Black arrows point to BrdU positive brown stained nuclei and arrow heads point to BrdU negative blue nuclei.

In an effort to establish the mechanism behind improved wound healing observed in the mice after the treatment of wounds with AGWJ, we aimed to determine if AGWJ treatment affected cell proliferation within the wound. To investigate this, we quantified the total number of cells in the center of the wound bed in high power field (20× magnification). The results did not show a significant difference in cell count between control treated (Supplemental Figure 2A) and AGWJ treated wounds (Supplemental Figure 2B) for the 5 day (Supplemental Figure 2C) time point. For the 7 day time point, the results observed for control treated wounds (Supplemental Figure 2D) and AGWJ treated wounds (Supplemental Figure 2E) also did not show a significant difference (Supplemental Figure 2F) suggesting that AGWJ does not affect cellularity of wounds. Several factors affect cellularity of the tissues including the rate of proliferation. We first measured cell viability *in vitro* in human fibroblasts by exposing the cells to AGWJ and the control media. The results did not show a significant difference in viability between AGWJ and control treated cells (Supplemental Figure 3). In order to verify the proliferation state of cells in granulation tissue *in vivo*, we also analyzed BrdU expression through IHC analysis. Positive BrdU protein expression was quantified as a brown stain in the nucleus of fibroblasts in the wound bed at the 5 and 7 day time points. Although the number of BrdU positive cells in the AGWJ treated group (Figure 2B) was slightly lower than the control group (Figure 2A) at 5 days post wounding (Figure 2C) and was higher than the control in 7 days post wounding (Figure 2F), the difference in results between AGWJ (Figures 2B,E) and controls (Figures 2A,D) were not significant. Therefore, collectively our results suggest that AGWJ does not enhance wound healing through modulation of cell proliferation in the wound bed.

### Unlike proliferation, AGWJ enhances fibroblast migration

**Figure 3:**
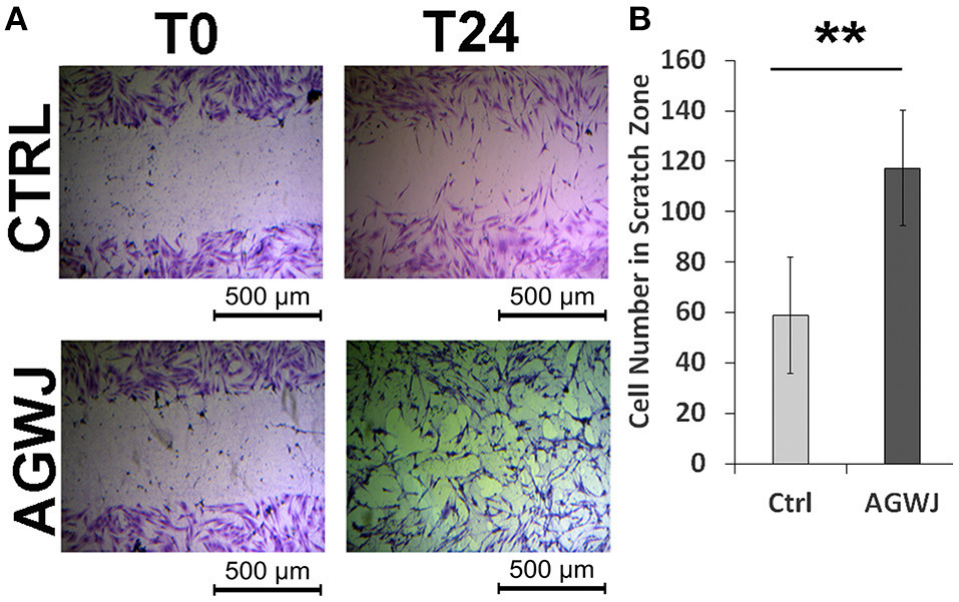
**Analysis of cell migration ***in vitro*** for fibroblasts. (A)** The top panel shows representative images of a control treated scratch at time point 0 h compared with the control treated scratch after 24 h. The lower panel displays the fibroblast scratch assay with AGWJ treatment at time point 0 h and then the scratch zone is imaged after incubation with AGWJ for 24 h. **(B)** The graph shows the average number of fibroblast cells that have infiltrated the scratch zone after 24 h for control media treated cells compared to AGWJ treated cells. ^**^Indicates the significance of *p* < 0.01 compared to control.

Since our *in vivo* wound analysis demonstrated that enhanced wound healing post AGWJ treatment was not due to cell proliferation in the wound bed, we investigated the possibility of enhanced cell migration causing faster wound closure. Cellular migration is an essential step during skin healing (Amini-Nik et al., 2011, 2014). To test the effect of AGWJ treatment on cell movement in wound healing, cell migration was analyzed *in vitro* by performing a scratch assay. Human fibroblasts were used as they are the main cellular component of the dermis. Two horizontal scratches were made in a confluent well of fibroblasts and either control media or AGWJ treatment was added. Images were taken at *t* = 0 h and *t* = 24 h (Figure 3A). After 24 h we quantified cell infiltration within the scratch zone and observed a significantly higher number of cells in the scratch zone for the AGWJ treated scratches as compared to the controls. For the control treated cells, an average of 58.85 ± 22.95 fibroblasts had migrated into the scratch zone, for AGWJ an average of 117.33 ± 22.86 cells migrated into the scratch (*n* = 3 control and *n* = 3 AGWJ, ^**^p < 0.01) (Figure 3B). Therefore, as shown in Figure 3A, the AGWJ treated fibroblast cells had started to close the wound after 24 h whereas the control cells had minimal migration into the scratch zone. Thus, AGWJ treatment of fibroblasts promotes their migration ability. We also observed that the AGWJ treatment altered cell morphology for fibroblasts. After 24 h the fibroblasts appeared to attain a more elongated phenotype as compared to the control media treated cells.

### AGWJ treatment promotes myofibroblastic phenotype *in vitro*

**Figure 4:**
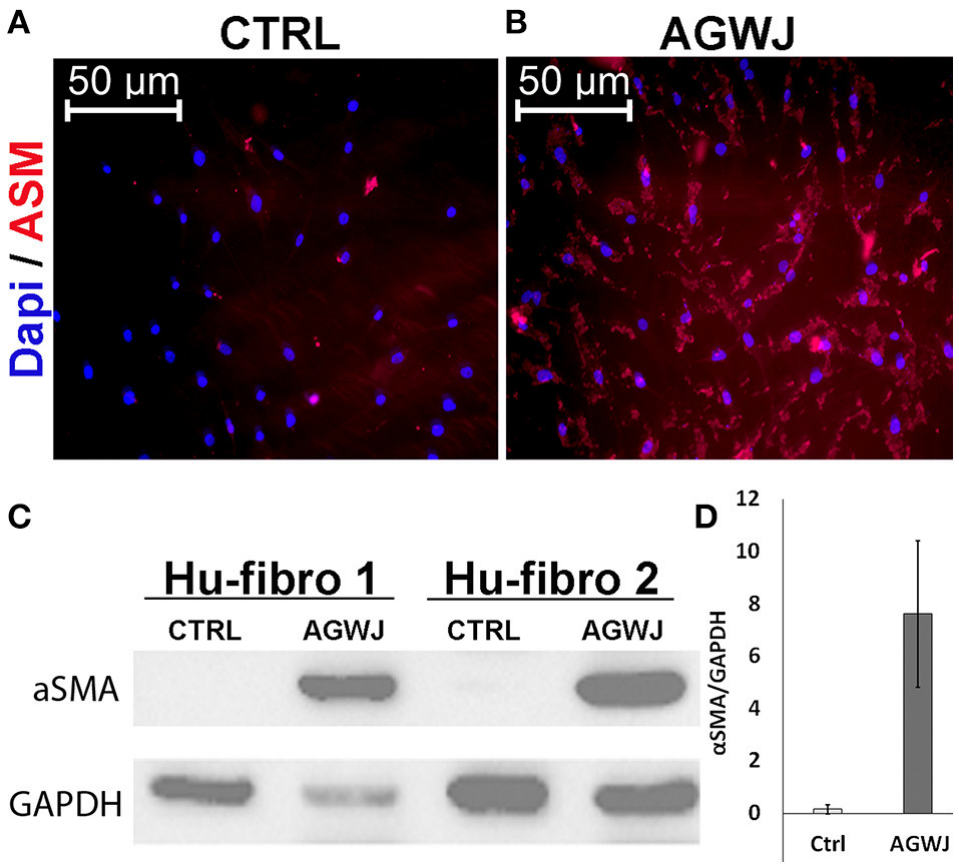
**AGWJ treatment promotes myofibroblastic phenotype ***in vitro*** detected through αSMA expression. (A)** Immunofluorescence image of control media treated fibroblasts compared with **(B)** AGWJ treated fibroblasts after 24 h of treatment. DAPI blue fluorescence represents nucleus and rhodamine red represents αSMA. **(C)** Western blot analysis of two normal human fibroblast cells (Hu-Fibro) treated with either control DMEM media or AGWJ treatment for 24 h in order to analyze αSMA protein expression. Loading control was GAPDH protein. **(D)** Densitometry analysis of the western blot. αSMA protein expression was normalized to GAPDH.

Since we detected a smaller wound size in the AGWJ treated wounds *in vivo* and our *in vitro* results demonstrated enhanced cell migration after AGWJ treatment, our findings support the notion that AGWJ augments cell migration. And that improved cell migration is the underlying mechanism for faster wound healing. Moreover, as mentioned, we observed a change in the phenotype of the cells treated with AGWJ *in vitro* and a smaller wound size *in vivo*, both of which suggest a pro myo-fibroblastic phenotype due to AGWJ treatment occurring earlier which led to faster contraction which is characteristic of enhanced wound healing. To evaluate this *in vitro*, we treated fibroblasts with AGWJ or control media and observed through immunofluorescence analysis that αSMA protein is increased dramatically in fibroblasts after AGWJ treatment (Figure 4B) as compared with control treated cells (Figure 4A). This result was corroborated by western blot analysis which detected a dramatically higher level of αSMA protein expression in AGWJ treated fibroblasts for two different human fibroblast samples (Figure 4C). The western blot band densitometry for αSMA was normalized to GAPDH and the quantification shows an almost 8-fold increase in αSMA protein levels after AGWJ treatment as compared with control DMEM media (Figure 4D). Furthermore, normal human fibroblasts display high levels of vimentin, which is reduced after AGWJ treatment for 24 h, observed in two different human fibroblast samples through western blot analysis (Supplemental Figure 4).

### AGWJ augments the differentiation of fibroblasts to myofibroblasts in the wound bed

**Figure 5:**
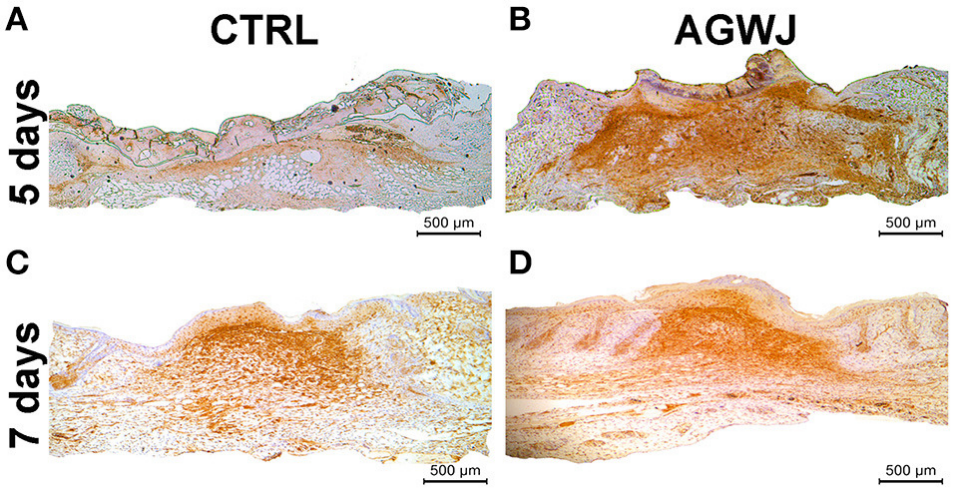
**Effect of AGWJ treatment on αSMA expression in the wound bed ***in vivo***. (A)** αSMA expression in the granulation tissue of control treated wounds after 5 days. **(B)** αSMA expression in the granulation tissue of AGWJ treated wounds after 5 days. **(C)** αSMA expression in the granulation tissue of control treated wounds after 7 days. **(D)** αSMA expression in the granulation tissue of AGWJ treated wounds after 7 days.

Because we detected an elevated level of αSMA in fibroblasts *in vitro*, which is a characteristic of myofibroblasts we wanted to investigate if this result is translated *in vivo*. Immunohistochemistry analysis of αSMA in wounds excised from the day 5 and day 7 time points demonstrate that for the 5 day time point; the AGWJ treated wounds express higher levels of αSMA (Figure 5B) whereas the control wounds express very little to no αSMA (Figure 5A). Surprisingly, in contrast, for the 7 day time point, we detected that the wound beds for both the AGWJ treatment (Figure 5D) and the control treatment (Figure 5C) both expressed high levels of αSMA. Hence, it appears that AGWJ accelerates the wound healing process through earlier fibroblast differentiation into myofibroblasts as seen at day 5, and therefore shortens the proliferation phase of skin wound healing.

## Discussion

There is a substantial amount of literature which investigate the beneficial effects of cellular therapy with WJ-MSCs, alone or within a scaffold (Zebardast et al., 2010; Azari et al., 2011; Shohara et al., 2012; Arno et al., 2014; Fong et al., 2014; Ribeiro et al., 2014; Sabapathy et al., 2014). Emerging reports are showing the promising role of decellularized biological matrices for tissue repair (Boccafoschi et al., 2015). Our group has reported that WJ-MSCs conditioned-medium has positive effects on wound healing in human *in vivo* in full-thickness skin excisional wounds on a mouse model through paracrine signaling via several mechanisms: upregulation of wound healing factors' gene expression, such as TGF-ß2, HIF-1α and PAI-1 in addition to enhancing human skin fibroblasts proliferation and migration (Arno et al., 2014). It is known that the niche or microenvironment of stem cells is crucial in supporting the MSCs and that there is significant cross talk and signaling that occurs between the cells and the environment (Boccafoschi et al., 2015; Khodadi et al., 2016). Moreover, there is the secretome of stem cells which is within the Wharton's jelly. In addition to being a reservoir of MSCs, Wharton's Jelly has been reported to be a great source of pro-angiogenic and wound healing promoting factors, such as IGF-1, TGF-ß1, VEGF, PDGF, EGF, bFGF, HGF, IL-6, and IL-8 (Sobolewski et al., 2005; Liu et al., 2012; Arno et al., 2014; Biazar, 2014; Edwards et al., 2014). These factors are also believed to stimulate the cells within Wharton's Jelly to produce large amounts of ECM components (Sobolewski et al., 2005). Because AGWJ is present in the native niche of MSCs and supports these stem cells, in addition to being a source of wound healing factors, we hypothesized that AGWJ has beneficial effects on skin wound healing. We, therefore, isolated the Wharton's jelly, and acellularized it (Supplemental Figure 1). In the umbilical cord, whether AGWJ promotes stem cell behavior and supports these MSCs in their niche or whether other cell types contribute to the microenvironment and consequently affects stem cell behavior is unknown.

The findings from this study established that AGWJ leads to healing at an earlier time point in a murine model demonstrated a significant reduction in wound length after AGWJ treatment as compared to control treatment after 5 days. We chose 5 days and 7 days as our time points for this study because, in a mouse model, day 5 is considered the peak of proliferation phase and day 7 is the start of the wound maturation phase in wound healing (Bielefeld et al., 2013). Despite the positive effect on wound healing of young animals that we report here, it is yet to be determined whether this effect can rescue deficient healing observed in elderly or diabetic patients. More research is warranted to address their effect on a deficient skin healing model.

We also tried to understand the mechanism of the enhanced wound healing through investigating several possible mechanisms, such as: cell viability, cell proliferation, and cell migration. We started by investigating cellularity in the wound by doing a cell count in the granulation tissue at HPF and also detecting cell proliferation through BrdU staining of the wounds. The results did not show any significant difference between AGWJ and controls. Therefore, our *in vivo* wound analysis suggests that enhanced wound healing post AGWJ treatment was not due to an increase in cell number. To further characterize this observation *in vitro*, we performed a viability assay using AGWJ and control media treatment. Cell proliferation can have an effect on cell viability and hence cellularity. Again, our data showed that AGWJ does not affect the viability of fibroblasts *in vitro*. Taken together, this set of data establishes that AGWJ's mechanism of action is not through enhancing proliferation, viability, and cellularity of cells.

We next investigated the possibility of enhanced cell migration causing faster wound closure. Cell motility and migration play critical roles during wound healing (Schneider et al., 2010; Amini-Nik et al., 2014). Indeed our *in vitro* results confirmed that AGWJ causes increased fibroblast migration. Moreover, we observed a change in the phenotype of the fibroblast cells treated with AGWJ and a smaller wound size *in vivo*, both of which suggest a pro myo-fibroblastic phenotype. IHC analysis of the wounds for αSMA showed an enhanced expression of αSMA in the granulation tissue after 5 days in comparison to control treated wounds. Surprisingly at the 7 day time point the αSMA expression in control and AGWJ treated wounds seems to be even. This suggests that AGWJ accelerates the proliferation phase of wound healing by causing a faster differentiation of fibroblasts to myofibroblasts confirmed through αSMA expression. The control wounds follow their normal course of differentiation and catch up later at 7 days post wounding. This result was supported by our *in vitro* analysis of fibroblasts post AGWJ treatment which showed an elevated expression of αSMA protein expression compared to controls.

This report shows for the first time that AGWJ enhances wound healing by accelerating the proliferation phase of skin healing mainly through enhancing cell migration and wound closure. Several diseases, including wounds in diabetic patients of elderly patients, are associated with delayed wound healing and AGWJ might be a remedy for this group of patients. Although we have found a mechanism through TGF-beta signaling pathway for the positive effect of AGWJ on skin healing, it is yet to be verified whether other factors that might contribute to this observation. Future research should focus on the further characterization of AGWJ and to find the active ingredients, particularly for the components of signaling pathways which have essential roles during skin healing, such as Wnt/β-catenin as well as TGF-β/Smad 2 signaling pathways (Amini Nik et al., 2007; Poon et al., 2009; Bielefeld et al., 2011). Since the AGWJ enhances wound healing mainly through cell migration and not cell proliferation, it might be an ideal remedy for the deficient skin healings which are associated with deficient migration. We have recently reported that elderly burn patients have reduced stem cell pool, a deficient migration of MSC and an altered activation of crucial signaling pathways for skin healing (Jeschke et al., 2015). Therefore, since our data suggests that AGWJ enhances cell migration, causes an earlier differentiation of fibroblasts to myofibroblasts and hence allows for earlier wound contraction, this treatment can have significant wound healing benefits, particularly for this group of patients.

### Economy of AGWJ

The findings from this study demonstrate the discovery of AGWJ which is the very native and niche material of stem cells within umbilical cords for the purpose of skin wound healing. We collect approximately 5 ml of jelly from each umbilical cord. To this 5 ml of jelly, we added 15 ml of DMEM complete media and re-suspended the jelly so that we had a total of 20 ml solution. For our *in vivo* study on mice, we placed 50 ul of AGWJ solution mixed with 50 ul of matrigel (1:1) on each wound, covering and enhancing 0.3 cm^2^ area of the wound in the animal. Collectively each umbilical cord provides AGWJ material for approximately 115 cm^2^ area of the wound (Supplemental Figure 5). Unlike the secretome of MSCs which needs an equipped facility to isolate, isolation of AGWJ needs minimal equipment and can be performed in any facility in developing countries.

In conclusion, we demonstrate for the first time that AGWJ enhances wound healing and establish a mechanism for its role as a potential therapeutic modality for deficient skin wound healing. Because each umbilical cord provides approximately enough AGWJ to cover 115 cm^2^ area of a wound, is easy to isolate and it is available globally; umbilical cord AGWJ has far reaching benefits for wound healing not just in the developed world but also in developing countries where affordable and available wound healing remedies are of critical need.

## Author contributions

NB, MJ, and SA have made substantial contributions to the conception and design, data acquisition, data analysis and interpretation for this study. NB and SA were responsible for creating the animal wound model and with the help of LM these authors were responsible for the organization, analysis and interpretation of the *in-vivo* data. NB performed the cell culture and *in-vitro* experiments. LM performed data analysis blinded. EH performed the surgeries to obtain the umbilical cords for the study. SA and NB contributed to the writing of the manuscript and performing revisions. All authors gave provided approval of this version of the manuscript to be published.

### Conflict of interest statement

The authors declare that the research was conducted in the absence of any commercial or financial relationships that could be construed as a potential conflict of interest.

## Supplementary material

Supplementary Figure 1 **Decellularization of Wharton's Jelly**. The degree of decellularization of Wharton's jelly was assessed using DAPI immunofluorescence staining nuclei. Panel one shows complete WJ before decellularization with many cells, panel two shows AGWJ without any cells.

Supplementary Figure 2 **Effect of AGWJ treatment on cellularity in the wound bed. (A)** Trichome stain analysis used to quantify the cell number in the wound center. **(A)** Representative image showing cellularity in the wound bed after 5 days of control treatment. **(B)** Image showing cellularity in wound bed post AGWJ treatment after 5 day time point. **(C)** Quantification of average cell number within the wound bed comparing control treated wounds with AGWJ treated wounds after 5 days. **(D)** The representative image displaying cellularity in the wound bed after 7 days of control treatment. **(E)** Image showing cellularity in wound bed post AGWJ treatment for 7 day time point. **(F)** Quantification of average cell number within the wound bed comparing control treated wounds with AGWJ treated wounds post 7 day treatment. The graph shows the average cell number in the wound bed of control compared to AGWJ treated mice under 20× magnification. Data shown are mean ± 95% confidence interval. For the 7 day study *N* = 7 for AGWJ treated mice and *N* = 6 for control mice, each n represents one animal. For the 5 day study, *N* = 3 for AGWJ and *N* = 3 for control treated mice.

Supplementary Figure 3 **AGWJ does not affect fibroblast viability *in vitro***. Quantitative analysis of cell viability post control and AGWJ treatment for 24 h. Luminescence was read using the Synergy H4 hybrid multi-mode microplate reader.

Supplementary Figure 4 **AGWJ treatment reduces vimentin expression in fibroblasts**. Western blot analysis of two normal human fibroblast cells (Hu-Fibro) treated with either control DMEM media or AGWJ treatment for 24 h. Loading control was GAPDH protein.

Supplementary Figure 5 **Schematic illustrating the isolation procedure for AGWJ and the subsequent economical use of AGWJ as a remedy for wound healing**.
