# Supplementary figures and images for: Acellular Gelatinous Material of Human Umbilical Cord Enhances Wound Healing: A Candidate Remedy for Deficient Wound Healing
Source: Front Physiol. 2017 Apr 4;8:200. doi: 10.3389/fphys.2017.00200 (PMC5379110; doi:10.3389/fphys.2017.00200)

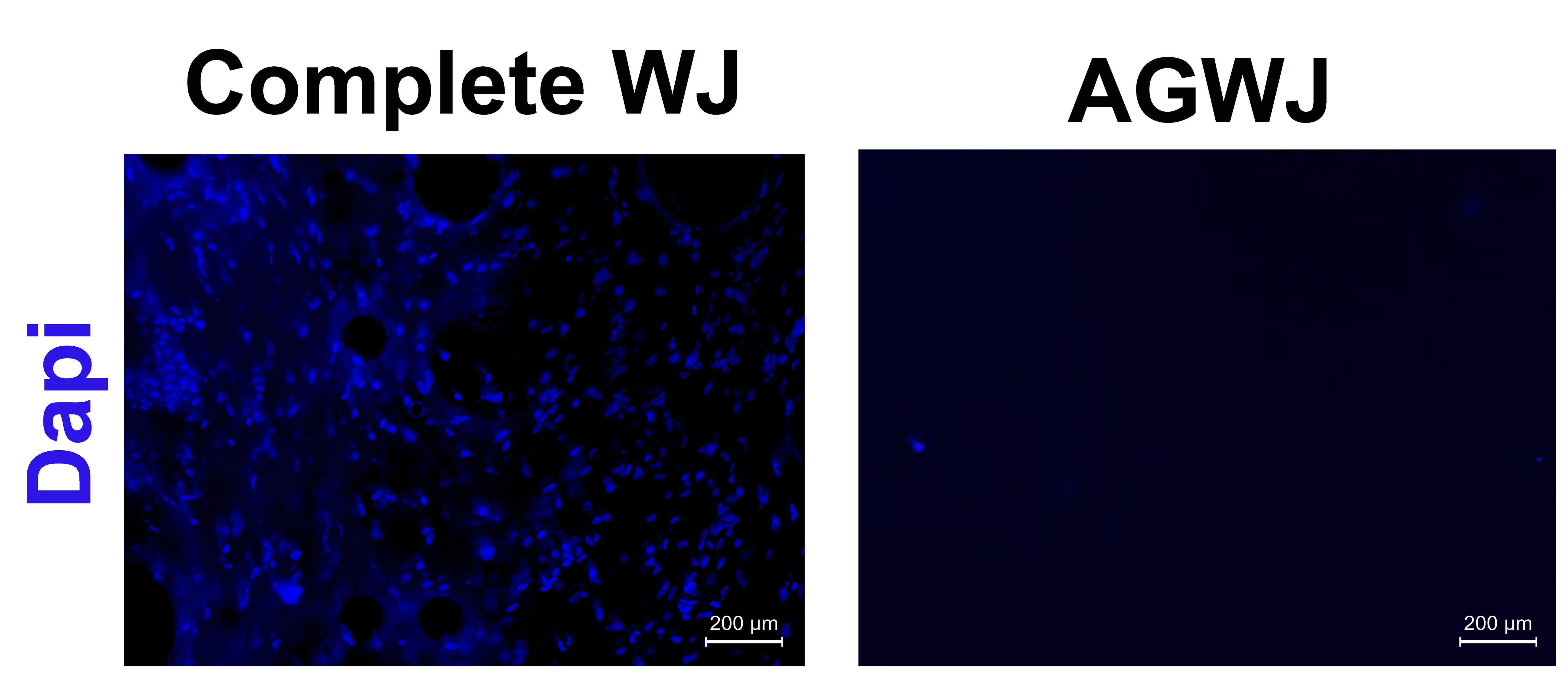

Supplement: Supplementary Figure 1 — Decellularization of Wharton's Jelly. The degree of decellularization of Wharton's jelly was assessed using DAPI immunofluorescence staining nuclei. Panel one shows complete WJ before decellularization with many cells, panel two shows AGWJ without any cells. [file Image1.tif]

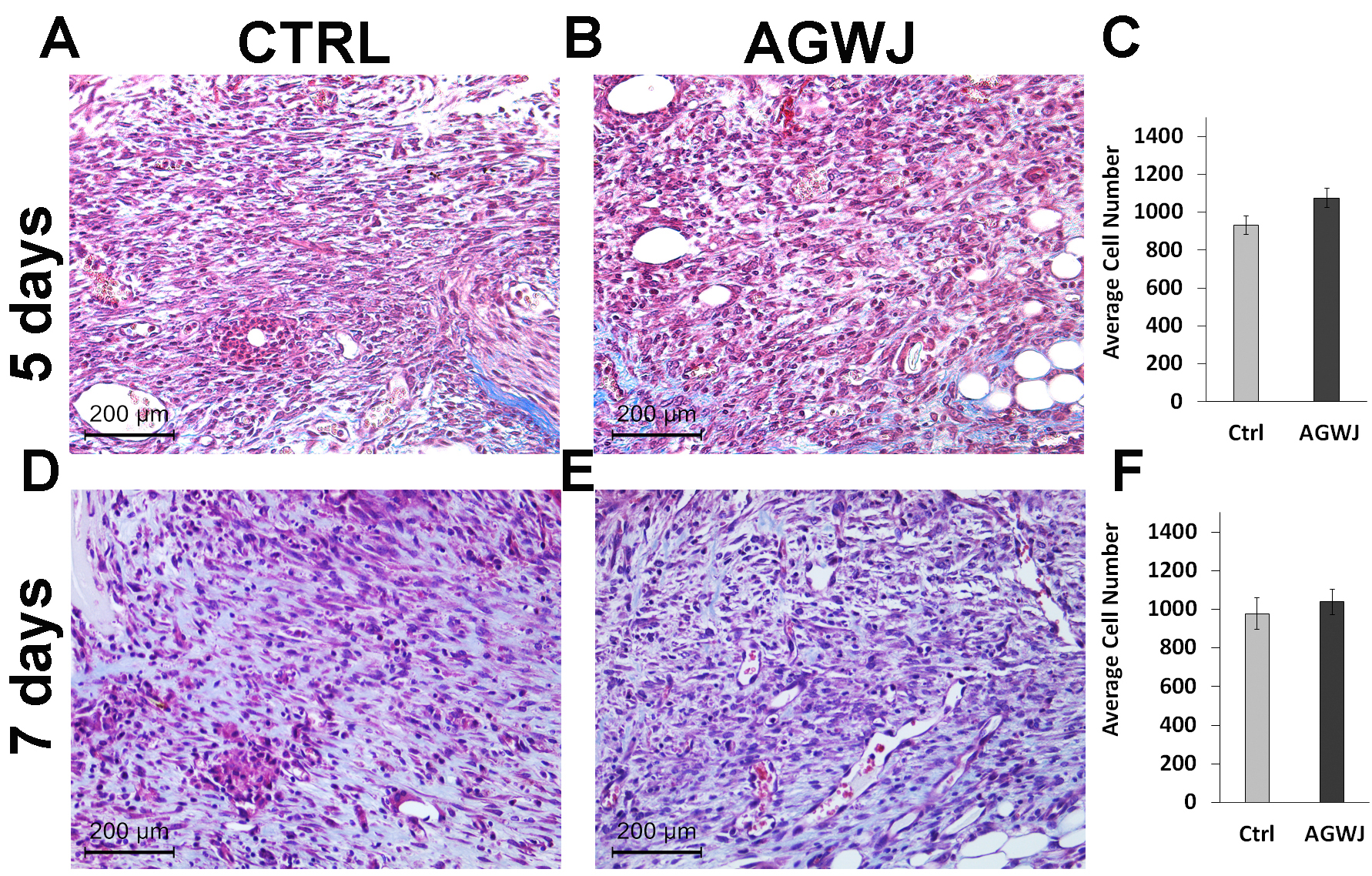

Supplement: Supplementary Figure 2 — Effect of AGWJ treatment on cellularity in the wound bed. (A) Trichome stain analysis used to quantify the cell number in the wound center. (A) Representative image showing cellularity in the wound bed after 5 days of control treatment. (B) Image showing cellularity in wound bed post AGWJ treatment after 5 day time point. (C) Quantification of average cell number within the wound bed comparing control treated wounds with AGWJ treated wounds after 5 days. (D) The representative image displaying cellularity in the wound bed after 7 days of control treatment. (E) Image showing cellularity in wound bed post AGWJ treatment for 7 day time point. (F) Quantification of average cell number within the wound bed comparing control treated wounds with AGWJ treated wounds post 7 day treatment. The graph shows the average cell number in the wound bed of control compared to AGWJ treated mice under 20× magnification. Data shown are mean ± 95% confidence interval. For the 7 day study N = 7 for AGWJ treated mice and N = 6 for control mice, each n represents one animal. For the 5 day study, N = 3 for AGWJ and N = 3 for control treated mice. [file Image2.jpeg]

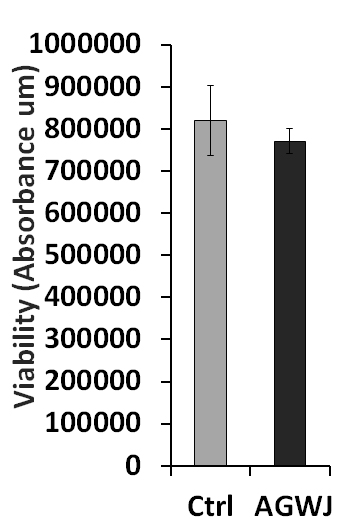

Supplement: Supplementary Figure 3 — AGWJ does not affect fibroblast viability in vitro. Quantitative analysis of cell viability post control and AGWJ treatment for 24 h. Luminescence was read using the Synergy H4 hybrid multi-mode microplate reader. [file Image3.jpeg]

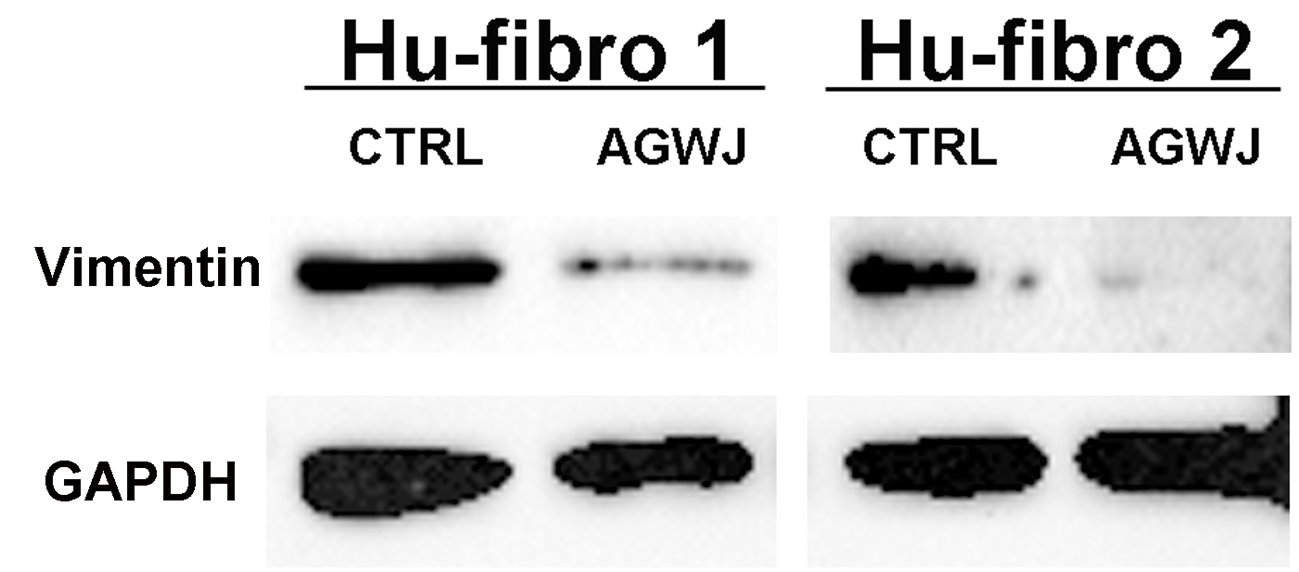

Supplement: Supplementary Figure 4 — AGWJ treatment reduces vimentin expression in fibroblasts. Western blot analysis of two normal human fibroblast cells (Hu-Fibro) treated with either control DMEM media or AGWJ treatment for 24 h. Loading control was GAPDH protein. [file Image4.tif]

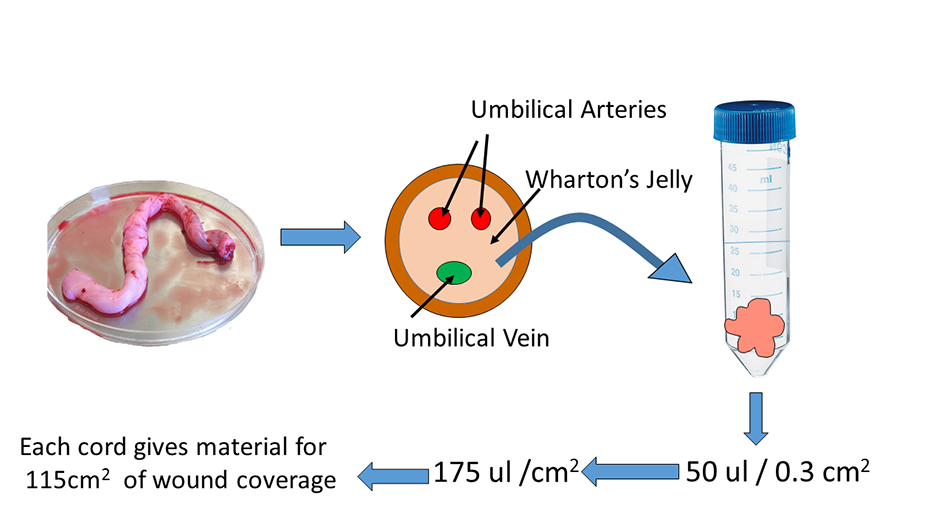

Supplement: Supplementary Figure 5 — Schematic illustrating the isolation procedure for AGWJ and the subsequent economical use of AGWJ as a remedy for wound healing. [file Image5.tif]
